# Blau syndrome mimics Takayasu’s arteritis: Report of 2 cases with literature review

**DOI:** 10.2478/rir-2024-0024

**Published:** 2024-10-21

**Authors:** Xin Ma, Bei Zhang, Wenjing Wang, Lindi Jiang, Xiaofei Shi

**Affiliations:** Department of Rheumatology and Immunology, The First Affiliated Hospital, and College of Clinical Medicine of Henan University of Science and Technology, Luoyang, Henan Province, China; Department of Rheumatology, Zhongshan Hospital, Fudan University, Shanghai, China

Dear Editor,

Blau syndrome (BS) is a rare autosomal dominant genetic disorder characterized by the clinical triad of granulomatous dermatitis, symmetric arthritis, and recurrent uveitis. The presentation of BS with vasculitis is exceedingly rare. In this letter, we describe two brothers diagnosed with BS, who presented with symptoms resembling Takayasu’s arteritis.

## Case 1

**Figure 1:**
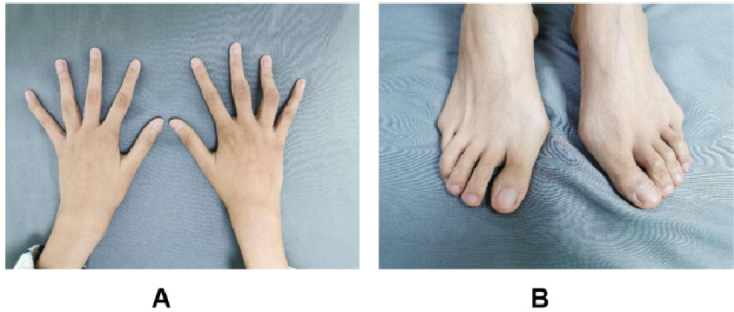
Joint manifestations of the case 1. A: swelling of the middle finger and ring finger of the right hand, ring finger flexion deformity, mild contracture; B: bunion of both feet and adduction deformities of his small toes.

**Figure 2:**
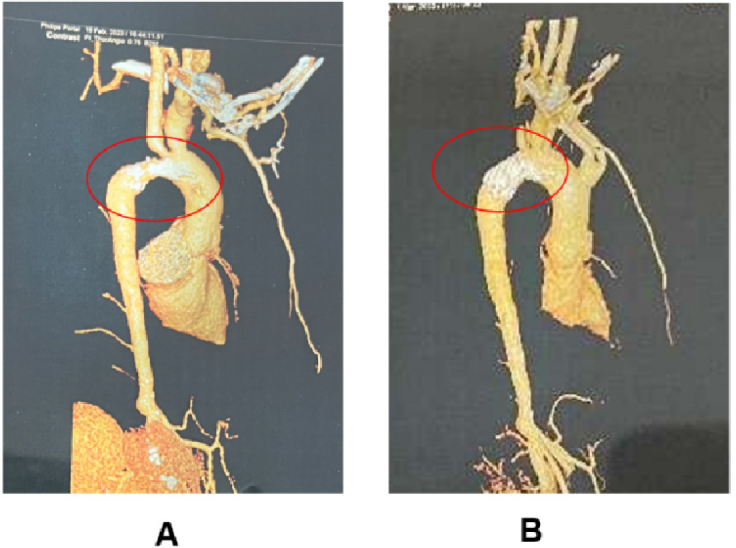
Aortic CTA of the case 1. A: Preoperative CTA of the case 1; B: Postoperative CTA of the case 1. CTA, computed tomography angiography.

A 17-year-old male presented with a decade-long history of joint swelling and pain, accompanied by hypertension persisting for over four years. He is the third child in his family, with a normal developmental history and no significant parental health issues. At the age of 12, he was diagnosed with rheumatoid arthritis following episodes of joint swelling and pain, and was subsequently treated with methotrexate and leflunomide. At 13, he began experiencing dizziness and discomfort, with his blood pressure measured at a high of 210/70 mmHg. Despite oral antihypertensive medications, his blood pressure remained poorly controlled. Upon his visit to us, he exhibited swollen joints in his hand with a mild flexion deformity in the right ring finger (Figure 1A), as well as thumb and toe deformities (Figure 1B). Laboratory tests, including blood count, liver and renal function tests, immunoglobulin levels, and complement levels, were within normal ranges, and anti-nuclear antibodies were negative. Imaging studies revealed a narrowed descending aortic arch and left ventricular hypertrophy. A positron emission tomography-computed tomography (PET-CT) scan demonstrated increased ^18^F-fluorodeoxyglucose (^18^F-FDG) uptake in the aortic arch and calcification areas, suggesting a possible diagnosis of Takayasu arteritis (Figure 2A).

## Case 2

**Figure 3:**
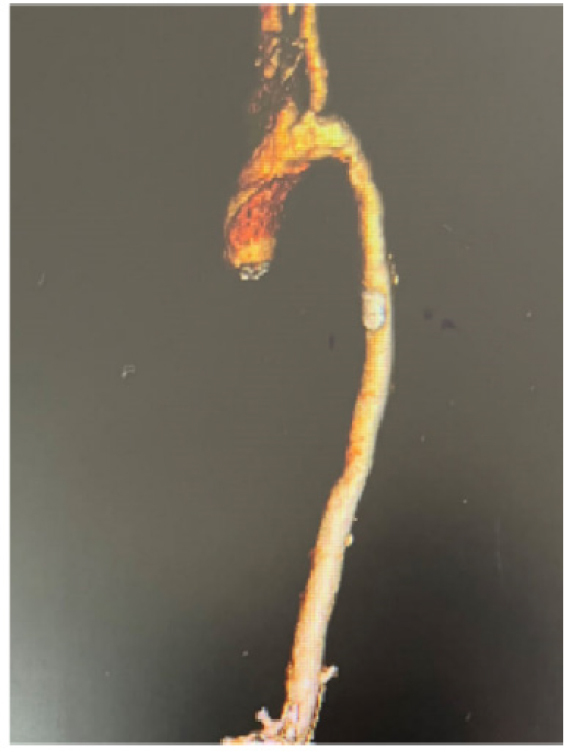
Aortic CTA of the case 2. CTA, computed tomography angiography.

A 19-year-old male presented to our outpatient clinic with right limb immobility, having been diagnosed with a cerebral infarction two years prior. He is the second child in the family and the elder brother of Case 1. Aortic CT angiography (CTA) revealed narrowing in the middle part of the aortic arch and other vascular abnormalities (Figure 3), initially leading to a diagnosis of Takayasu arteritis.

Whole-genome sequencing of the family revealed that all three brothers carried a heterozygous variant in the nucleotide oligomerization domain 2 (NOD2) gene. Mutational analysis identified a c. 1759 C > T mutation in all three siblings, while the parents showed no NOD2 gene variants. Consequently, both patients were diagnosed with BS. The oldest brother underwent a vascular ultrasound, which showed no abnormalities. Case 1 received treatment with adalimumab combined with methotrexate. Balloon dilation and intravascular stenting were performed on the stenosed arteries (Figure 2B), normalizing his blood pressure. Pathological examination of the anterior wall of the ascending aorta revealed mucinous degeneration and inflammatory cell infiltration, confirming inflammation. Despite medical treatment, Case 2 experienced sudden cardiac death one year later.

BS is a rare granulomatous auto-inflammatory disease caused by NOD2 gene mutations.^[1]^ With age, most patients suffer severe complications such as blindness, joint damage, and visceral involvement. In the absence of uveitis or skin rash, patients with initial arthritis symptoms are often misdiagnosed with juvenile idiopathic arthritis. Other internal organs can also be affected. Common manifestations include fever, hepatosplenomegaly, lymphadenopathy, and involvement of the heart, central nervous system, kidneys, and blood vessels. BS with vasculitis is rare, with the middle part of the aorta, renal artery, and cerebral artery most commonly involved and typically asymptomatic.^[2]^ Zeng reported a 20-year-old girl initially misdiagnosed with adolescent idiopathic arthritis who developed renal arteritis at 17 and was later diagnosed with BS due to a heterozygous NOD2 mutation.^[3]^ Cardiovascular lesions in BS patients are often identified when hypertension develops during follow-up, caused by large-artery vasculitis.^[4]^ The two cases we report presented with unusual BS presentations mimicking Takayasu’s arteritis. A study of 38 Chinese children with BS revealed that approximately 34.2% developed Takayasu-like vasculitis and cardiopathy.^[5]^

Due to the rarity and complex clinical manifestations of BS, there is a lack of large cohort research on treatment effects. Specific treatments for BS have not been fully determined.

Glucocorticoids and immunosuppressants, such as azathioprine and methotrexate, may improve prognosis. Biologics, including tumor necrosis factor inhibitors, tofacitinib, and tocilizumab, have shown promising therapeutic effects,^[6]^ but further studies are needed to confirm these findings.

In summary, we described two male patients from the same family initially diagnosed with Takayasu’s arteritis and later confirmed with BS through genetic testing. This study highlights the importance of genetic testing when patients present with skin rash, arthritis, or a family history. Rheumatologists and pediatric rheumatologists must be aware of these rare clinical manifestations. Regular physical examinations, including pulsation checks and blood pressure measurements, may aid in early diagnosis. Vascular imaging should be performed to assess the degree and extent of vascular damage, as early diagnosis and appropriate treatment can prevent or delay disease progression.

As an autosomal dominant disease, genetic testing is crucial for diagnosis. In our cases, neither parent exhibited genetic abnormalities, yet all three children carried the NOD2 variant, with two developing the disease. This suggests potential gonadal mosaicism in the parents. Germline mosaicism, where a gene mutation is present in some germ cells but not in somatic cells, can result in the production of gametes with disease-causing genes, which can be inherited by offspring. In such cases, DNA from sperm and egg cells should be tested for mutations. However, this was not performed as the parents of these two cases declined genetic testing for personal reasons. As there are no reports of germline mosaicism in BS patients in the literature, There are no reports of germline mosaicism in BS patients in the literature, so future studies should investigate this hypothetical mutation.
